# Crossing the wicked frontier. Why sustainability science needs integrative research

**DOI:** 10.1098/rsos.240210

**Published:** 2024-09-04

**Authors:** Richard J. Hewitt

**Affiliations:** ^1^ Institute of Economy, Geography, and Demography, Centre for Human and Social Sciences, Spanish National Research Council (CSIC), C. de Albasanz 26, 28037 Madrid, Spain

**Keywords:** integrative research, wicked problems, post-normal science, science–policy interface, sustainability, climate change

## Abstract

Integrative research approaches have lost prominence in the scientific literature, and related concepts of ‘wicked problems’ and ‘post-normal science’ complement but do not adequately replace them. From a detailed examination of the foundational literature, three key principles are shown to be central to integrative research: (i) the knowledge cycle; (ii) representativeness and participation; and (iii) knowledge exchange mechanisms at the science–policy interface. As an example of the importance of the integrative research framework in the context of policy-relevant science, it is argued that the shortcomings of current climate change mitigation efforts arise from inappropriately ‘closing down’ the science–policy interface and focusing on a few narrow options acceptable to powerful stakeholders. This can lead to what is described as an ‘ascientific’ ratchet effect, where the knowledge cycle just loops endlessly between technology and capital, and science as a public good is excluded. An integrative research approach can ‘open up’ discourse to new ideas and actors and restore the iterative links between science–policy mechanisms. A theoretical framework is proposed in which the three concepts ‘wicked problems’, ‘post-normal science’ and ‘integrative research’ are nested together. Integrative research is a way to address wicked problems, sitting within the critical framing of post-normal science.

## 1. Introduction

Modern sustainability science is an awkward mix of knowledge communities [1]. As with human society in general, which it necessarily reflects, the practice of science is shifting and impermanent, and fixed, essential truths are few, if indeed they exist at all. At any given moment, some communities and their perspectives will be strongly dominant in public discourse, while others may be virtually invisible. This process can be viewed as a kind of ‘survival of the fittest’ natural selection of scientific knowledge [2,3], or from the perspective of a competitive ‘marketplace of ideas’ [4]. Yet the evolution of scientific knowledge is a complex process which both shapes and is shaped by society [5]; some good ideas and successful ways to approach problems also frequently get replaced with ideas that are not as good, or that are simply different, for reasons that may not be immediately clear. In the case of ideas and approaches which tackle questions of great urgency or social importance, such as global biodiversity loss or the climate crisis, it is worth asking what is lost through this process. We may wonder, when faced with the disappearance of a once celebrated concept or approach, if its loss is more closely related to some perceived inconvenience to particular social actors, or its lack of alignment with dominant currents of thought, than to any natural evolution of scientific knowledge. With the passing of time, new circumstances emerge, the gaps left by these earlier, now part-forgotten or marginalized concepts become ever more visible. Integrative research [6] is one such concept that has left one such gap; it is the focus of this piece.

The following article investigates this once popular, now almost invisible concept, proposed in the late 1990s and refined in various constructive ways up until around 2010, as a useful way of describing and structuring commissioned research for tackling difficult environmental problems. The case is a peculiar one: despite some strong successes in the application of integrative research as a conceptual framing for policy-relevant environmental science, the concept, at least in the sense intended by its original practitioners, has mostly disappeared from the literature. Once again, when working on large projects with specialists from different fields, we are confused about whether our work is ‘interdisciplinary’, ‘transdisciplinary’ or ‘multidisciplinary’, concerns that the integrative research framing had seemed to satisfactorily resolve [7,8]. However, the goal of this paper is not to discover why this useful and practical framing has fallen out of view in the environmental science literature, but to dig it up again, and expose it to the light. The aim in so doing is to show that integrative research, far from being a dead-end, is a seed that has not yet germinated, and is fully and vitally relevant to the present and future practice of science.

## 2. Integrative research and knowledge systems in science and policy

Integrative research, which emerged, in the sense used in this paper, out of large-scale European Union-funded research projects in the 1990s, is a way to negotiate compromises across knowledge communities—as distinct from scientific disciplines^1^—that allows researchers ‘to construct a composite knowledge system that capitalizes on perceived strengths without compromising the logical consistency of the whole’ [7]. Integrative research recognizes that knowledge systems are composite belief systems in which individual practitioners tend to hold conceptual framings which exclude ways of looking at problems outside their own domain. This leads to a tendency for each knowledge community to push ill-posed problems (also called ‘wicked problems’ [9]) beyond the limits of its domain. In this scenario, there is a rush to solve the easy problems, creating a kind of bubble economy around trendy new ideas which are easily tackled with existing methods. It is interesting to contrast this view of research with the vision of Thomas Kuhn [10], who saw science as a series of dramatic shifts of understanding (paradigms), provoked by crises of belief in earlier ways of viewing the world. In between these critical transitions, according to Kuhn, science explored problems within the boundaries of accepted ideas, navigating a kind of metastable plateau, until the existing system (paradigm) became unstable again, tipping into a new metastable state. Kuhn’s insight into the development of modern scientific disciplines from earlier philosophical currents (natural philosophy = science) shows the boundaries of knowledge communities as fluid and continually evolving; however, his work was strongly historical and explanatory, and he did not go so far as to advocate a framework for understanding and linking knowledge systems. Post-normal science (PNS; e.g. [11]) defines itself in opposition to the Kuhnian universe, but in fact builds on Kuhn’s recognition of the fluidity of boundaries between ideas. According to Funtowicz & Ravetz [11], ‘The reductionist, analytical worldview which divides systems into ever smaller elements, studied by ever more esoteric specialisms, is being replaced by a systemic, synthetic and humanistic approach’ [11]. The contribution of integrative research is to provide such an approach [12]. Without deliberate efforts to force and structure boundary crossing activities, the brave new world announced by [11] is replaced by a world in which the boundaries of knowledge domains are never explored, and Kuhnian ‘paradigm shifts’ cannot take place. Knowledge communities grow risk averse, throw up defences, and integration, in its fullest sense of unification of research across knowledge communities, cannot happen.

When this occurs at the science–policy interface, problem-solving collapses into stereotypical positions pushed by the most powerful stakeholders and dissident knowledge communities are shut out of the dialogue. In climate policy, and in mainstream discourse around climate action to which it is closely linked, this has already occurred. For example, there is an enormous interest from governments and the general public in afforestation as a mitigation tool (against a much lesser emphasis on regeneration and ecosystem restoration) [13], electric vehicles for reducing emissions in cities (rather than promoting public transport or reducing the number of private vehicles) [14] and low carbon energy solutions for new buildings (rather than limiting urban development or reorganizing cities to reduce travel) [15]. This is but a small selection of the numerous other ‘comfortable’ or incrementalist discourses that are palatable to modern societies, and unfortunately, much more strongly represented in public discourse on climate action generally than they probably deserve. Data scientist Hannah Ritchie’s recent book *Not the End of the World* [16] offers an example of this kind of thinking. Ritchie claims to provide an upbeat view of the challenges presented by the climate crisis. However, her book criticizes degrowth as unrealistic without seriously engaging with the concept, suggests that low-income countries will naturally gravitate towards clean energy solutions simply because of affordability, and professes to be ‘apolitical’, while advocating solutions based on markets and individual choice, positions usually associated with mainstream ‘liberal’ economics and the political right. The difficulty of this perspective, which is often shared by governments, is that it does not address the real question of how to move the relationship of human societies with their environment onto new, less harmful trajectories. Though there is certainly a need to remain optimistic, policies that move the world in this direction, with very few small-scale or partial exceptions (e.g. [17,18]), have yet to be implemented, and it does no one any good to pretend otherwise. In this sense, Antal *et al.* [19] have argued that the sustainability literature needs a stronger focus on negative (i.e. environmental unsustainability) rather than just positive trends. At least in part, the role of science must be to help us swallow the medicine, even if it is nasty. Without research across knowledge communities, the gaps in discourse, and by extension, in research and policy, are not identified or acted on.

The main aim of this paper then, is to examine the integrative research concept in detail, to see how it can usefully be applied to today’s most pressing environmental sustainability concerns. At present, there is no single clear framework for integrative research and some of the key literature is rather philosophical in tone (e.g. [6]). However, several important key principles, which deserve much more attention and visibility than they currently have, do emerge very clearly from a closer reading of the literature.

This article is presented in two parts, following a literature review and analytical discussion approach. In the first part of this article, to extract these key principles and show how they may be operationalized, I review publications which are either specifically about integrative research, or which clearly reference integrative research as a key theoretical foundation or approach. Relevant literature was identified through a keyword search in Google Scholar and Web of Science, and results were screened for relevance to the central theme of this article, environmental research at the interface between science and policy.

In the second part of the article, I discuss more recent literature from the sustainability science community on ‘wicked problems’ and ‘PNS’, in order to show how integrative research provides the structure that is missing from these theoretical positions. The main contribution I hope to make is to show that these three concepts belong together in sustainability science, that they are of the same family and that integrative research is their orphaned relative who needs to be brought back into the fold. I argue that the absence of efforts to effectively bridge knowledge communities, and bring different perspectives to bear on each of these discourses leads to repetitive thinking and one-sided messaging, depriving society of important knowledge about their key limitations. Integrative research perspectives on all three discourses allow the discussion to be *opened up* [20,21].

## 3. Key themes from integrative research literature

To extract the most important literature, we followed the definition given above, of integrative research as research which systematically works across coherent knowledge communities with different belief systems and involves direct participation from representatives of each. This means that research which aims to link different concepts (e.g. [22]), different research approaches (e.g. [23]) or different technical specialisms (e.g. [24]) within the same knowledge community falls outside this definition. I also do not include research that unites different elements or disciplines through literature review or simply provides an integrating framework without direct engagement from different stakeholders (e.g. [25]). This is not to suggest that these kinds of studies are not useful in their own right. However, it is this effort of bridging different belief systems, including the active engagement of members of separate knowledge communities that gives integrative research its special power to tackle ill-posed or wicked problems like the climate crisis.

The majority of the references found under this definition broadly belonged to the field of environmental management and allied domains like agricultural science or landscape studies. Though the words ‘integrative research’ were found outside this broad science domain, they usually referred only to integration of different conceptual approaches, often in reference to literature-based research only. It seems that only environmental scientists considered that integrative research should link directly to practitioners or policy makers or involve non-academics in participatory knowledge development [8,26]. The specific sense of integrative research as an activity linking knowledge communities and different belief systems, with representatives of these separate communities as active participants, does seem to be unique to the field of environmental science and policy.

Table 1 presents the key literature on integrative research in chronological order. Most of the papers cited at least one of the other listed references, and many members of this small community have worked together. However, despite the small size of the community, some of the papers are well cited, suggesting some degree of influence beyond the group. We discuss this literature briefly as follows in order to highlight its main themes and evolution. The term ‘integrative research’, in the sense we use in this piece, seems to have emerged in the mid-1990s, with a paper by Park and Seaton in the journal *Agricultural Systems* [12]. These authors called for discipline-based research around sustainability to be explicitly linked with policies to implement it through a series of ‘decision interfaces’ [12]. At each decision interface, different groups from different specialist knowledge communities must interact to keep the science-to-policy process adaptive and flexible. This helps to ensure, for example, that farmers’ own evaluations of the feasibility of agricultural policies are actively included even when they contradict advice given by scientists [12]. Winder [7,8] adopted a knowledge systems perspective to develop the integrative research concept as a specific and much needed antidote to disciplinary isolationism, and especially as a force for pragmatic deployment of policy-relevant research on large-scale applied research schemes like the EU Framework programmes. Crucially, Winder did not separate the academy from society at large, and talked of knowledge domains and knowledge communities rather than scientific disciplines, recognizing that many non-scientific stakeholders (e.g. farmers, as discussed by Park & Seaton [12]) are key knowledge providers, not passive recipients. Tress *et al*. [8,27] formalized the definition of integrative research [8] and analysed the barriers to research across disciplines in the field of landscape studies [27]. These authors introduced the fundamental idea of the ‘cycle of knowledge’ where different disciplinary communities work together to solve a specific problem, leading to new knowledge or insight, which is then fed back into the system, and the cycle begins again [8]. These authors discussed the role of non-academic participant stakeholders, who may cooperate with researchers in different ways, through knowledge transfer (one-way), exchange (two-way) or integration (stakeholders influence project goals, execution or outcome) [8]. Macleod *et al.* [21] obligingly demonstrated the knowledge cycle in practice exactly as Tress *et al*. [8] described, by synthesizing the previously discussed literature in the context of Stirling’s [20] influential paper on the role of participation in environmental science and policy. By incorporating Stirling’s [20] structuralist vision of the science–policy interface, through iterative cycles of opening up and closing down, [21] showed how integrative research allows environmental policy to be implemented in practice. In calling for a policy process that prioritizes coproduction of knowledge, such that ‘policy makers and potential users [are] involved in a two-way dialogue from conception’, [21] echoed the strategy set out by Park & Seaton [12] over a decade earlier. At this point, integrative research, in the sense described by this paper, was conceptually complete. References [26] and [28–32] provide case studies, which usefully illuminate the practical application of integrative research, and are discussed in detail in later sections of this paper.

**Table 1.** Integrative research literature and its key themes.

key literature on integrative research	summary	key themes	source
Park & Seaton (1996) [12]	integrative research to link different areas of research and practice to improve sustainability of agriculture	the construction of ‘research interfaces’ to link disciplines (horizontal integration), and connect science and policy (vertical integration)	agricultural systems
Winder (2003) [7]	definition of integrative research as ‘research across [knowledge] communities’, synonymous with ‘interdisciplinary’ and ‘transdisciplinary’ approaches but involving non-science stakeholders	knowledge domains rather than disciplines, ill-posed problems, history of knowledge systems	in: interdisciplinary and transdisciplinary landscape studies: potential and limitations
Winder (2005) [6]	examines the practice of research projects from an integrative research perspective, emphasizing the value of process. Discusses the tension between modern research programmes (e.g. the European Union Framework Programmes 5 and 6) that cut across sectors and disciplinary boundaries and institutions in which those projects are executed	appreciation as a discursive process by which judgments are made to form policy. The difference between reality judgments, operational judgments and value judgments. The implications of the difference between Mode 1 (teaching and scholarship) and Mode 2 (project research) in institutions	systems research and behavioural science
Tress *et al.* (2005) [8]	systematic definitions of integrative research for landscape planning	‘knowledge cultures bridged and their knowledge fused together’. Collaborative work between researchers and non-researchers. The circle of knowledge creation	in: from landscape research to landscape planning: aspects of integration, education and application
Tress *et al*. (2007) [27]	analysis of the barriers to integration in landscape research projects through semi-structured interviews and Web survey	identify the problem of time management as the most significant barrier, followed by a lack of common terminology and differences in academic traditions across disciplines	land use policy
Macleod *et al*. (2008) [21]	lessons learnt in the development of an evidence base for policy makers to support sustainable catchment management. Identifies ‘opening-up’ mechanisms (the Integrating Water and Agricultural Management collaboration between scientists and policy makers) and ‘closing-down’ mechanisms (the Diffuse Pollution User Manual). Recommends that opening-up and closing-down mechanisms should be explicitly connected through iterative back and forth processes of knowledge exchange	integrative research spanning researchers and policy makers. Explicitly links the ‘opening-up’ and ‘closing-down’ distinction of Stirling [20] to integrative research for environmental policy	ecology and society
Cartledge *et al*. (2009) [28]	integrative approaches to action at the science–policy interface	constructive ambiguity, opening up (problem-framing), closing down (problem-solving)	ecology and society
Hernández-Jiménez & Winder (2009) [29]	describes integrative research in environmental planning in a rapidly urbanizing region with weak development control (Madrid). Identifies a strategy of ‘paralysis by analysis’ whereby powerful actors encourage noisy discourse but avoid compliance with sustainability objectives	opening up discourse at the science–policy interface as a deliberate disruption mechanism. Working for sustainable development through back and forth analytical/discursive research activities	in: beyond the rural–urban divide: cross-continental perspectives on the differentiated countryside and its regulation
Kragt *et al*. (2013) [30]	environmental modelling to facilitate knowledge integration across academic communities	models to bring together diverse researchers with different epistemologies, methods, procedures, concepts and theories	environmental modelling and software
Priess & Hauck (2014) [31]	participatory development of land use scenarios with researchers and practitioners	integration across the science–policy interface with large numbers of practitioners (~160). The work was able to capture assumptions, demands and perspectives in one set of regional scenarios, and ensured salience for the intended users	ecology and society
Hewitt *et al*. (2017) [26]	participatory approaches to understanding renewable energy deployment	power relations and interaction between actors from different interest communities, stakeholder diversity, stakeholder representativeness	energy research and social science
Hewitt *et al*. (2017) [32]	tools, methods and case studies for building participatory solutions to human–environment interaction problems with stakeholders	integrative research as a four-stage cycle of actions integrating discursive and analytical activities (*sensu* 20])	participatory modelling for resilient futures: actions for managing the environment from the bottom up

This foundational literature, as discussed above, sets out a series of key principles, as follows:

- the knowledge cycle;
- representativeness and participation; and
- knowledge exchange mechanisms.

### 3.1. The knowledge cycle

**Figure 1.**
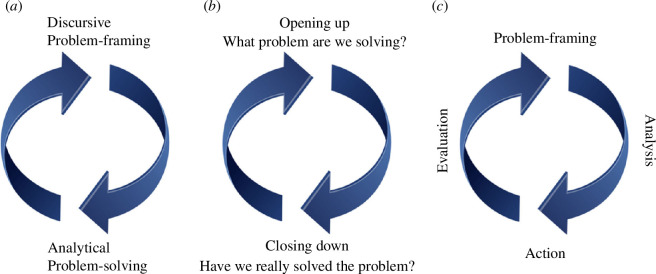
Three conceptualizations of the knowledge cycle: (*a*) as back-and-forth activities between discursive problem-framing and analytical problem-solving (after [29]); (*b*) mechanisms across the science–policy interface (e.g. [20,21]); (*c*) four actions integrating discursive and analytical activities at any point (after [32]).

The first key principle of the integrative research approach is the knowledge cycle or knowledge circle [8] (figure 1). An integrative research project involves interaction between different knowledge communities around the solution of a specific problem (the project), which may be more or less clearly defined, but is always present in some form, for without it the research activities are not directed towards any concrete outcome. It may happen that the intended outcome is found not to be achievable, and depending on when this is discovered, the project may refocus in order to solve a different problem. This has been called ‘constructive ambiguity’ [28]. Tress *et al.* [8] describe how these attempts to solve a specific problem (specific knowledge) are fed out to the wider community (generic knowledge), which in turn influences the approaches, concepts and tools employed in solving new problems (figure 1). The knowledge cycle can be understood in different, though closely related ways. Introducing the concept, Tress *et al.* [8] envisaged a cycle of specific problem-solving, generating new generic knowledge, a process that, according to Hernandez-Jimenez & Winder [29] necessarily involves cycling between participatory (discursive) problem-framing activities and analytical scientific activities designed to solve them (figure 1*a*). This process mirrors the conceptualizations of Stirling [20] and Macleod *et al*. [21] of an iterative sequence of opening-up and closing-down activities across the science–policy interface (figure 1*b*). The process can be further subdivided to emphasize the importance of concrete stages in the knowledge cycle [32], from problem-framing, through analytical activities, to implementation of specific actions, and finally, evaluation of outcomes, before the results and lessons learnt are fed back in to adjust or reframe the existing problem or scope out new ones (figure 1*c*).

A number of observations can be made. Firstly, we see that if the specific knowledge generated around a key problem is not fed back out to create generic knowledge or does not return to society in a usable or understandable way, this hinders new problem-solving activities. This relates to Kuhn’s ideas [10] around the breakdown of a scientific paradigm—under the knowledge cycle approach, the cycle stops turning once specific problem-solving efforts cease to provide knowledge that is useful to society. However, unlike in a Kuhnian world, society does not passively await the emergence of a new paradigm from practitioners of a remote activity called science. Since views around what is useful to society are diverse, and subject to political influence, the knowledge fed back out to society is filtered, simplified and synthesized (by politics, by the publishing industry, by the media), later feeding back into the system and influencing, even determining, the specific problems that can be tackled.

A second point to make is that the knowledge cycle is an integrative research cycle, in which representatives of different knowledge communities and belief systems interact to solve a common problem [8]. This intentionally disrupts the idea of science as an objective, elite activity that can be separated into individual silos; when it relates to matters of such great general concern as the climate crisis—it is not just climatologists who need to make the case for a transformational societal response. The integrative research cycle makes this quite explicit—for a broadly useful and effective science of climate action, the inclusion of representative groups from all domains in the knowledge creation process is essential (e.g. [7,12,26,31]). While there is certainly huge scientific interest in the climate crisis from many different fields and walks of life, the proposals for climate action that emerge are selective. Why is this, and what good ideas are we missing? Followed to its logical conclusion, the knowledge cycle could become a downward ratchet, where global interest in ever-fewer ideas leads to an ever-greater proportion of research effort expended on this ever-smaller pool, with resulting diminishing returns for science, society and for climate action.

### 3.2. Representativeness and participation

The next key principle of integrative research that emerges from this literature is that it aims to be *representative* of diverse methods, diverse stakeholder communities and diverse ways to view a problem [6,29,31]. This arises out of a critical response to positivist notions of absolute objectivity, as well as a recognition that many stakeholders (and hence their discourses) are marginalized in many conventional social interactions. A key example of this arises in written sources of evidence, often a researcher’s first port of call for understanding a new issue or field of study. For example, Goodwin [33] has argued convincingly that the key innovations in transport practice and policy are not found in academic literature, and that the increasing visibility and status accruing to journal articles and citations to them in the transport field does not reflect their rather low real impact on the field, compared with government transport briefings, special committee reports or policy papers. These latter, essential contributions are sidelined in academic literature, giving rise to academic discourses that are disconnected, impractical and narrow. This is also true of the community energy field, which has a burgeoning academic literature, but cannot be properly understood without detailed study of media reports and community websites which are the original source for this information (e.g. [34]).

The most important way to ensure representativeness is by incorporating divergent discourses from separate knowledge communities in the knowledge generation process itself through *participation*, the mechanism by which the knowledge exchange between the different communities is enabled. Participatory engagement with key actors having knowledge or interest in an issue or challenge (‘stakeholders’) is a thus a key part of integrative research. Kragt *et al*. [30] state this clearly, defining an integrative model as a process of negotiation between different parties. Hewitt *et al*. [35] take a similar approach showing how an integrative environmental model acts to integrate ‘soft’ (social sciences) and ‘hard’ (natural science) approaches around a common problem. However, participatory approaches are nowadays broadly included in many research processes which do not define themselves as integrative. A Delphi process developed in an ‘expert’ workshop is participatory, but it may not be integrative since it does not try to bring diverging views to the table. It is the attempt at representativeness, and the honest declaration of the difficulty of achieving it where it is not possible, that is the hallmark of the integrative approach.

Indeed, often it may not be possible to ensure representativeness within a stakeholder group. This was the case of Hewitt *et al*.’s [26] analysis of links between entities involved in renewable energy development in Navarre, Spain, in 2013. Despite, or perhaps because of, the great importance of the business sector, which, in Spain, led the development of renewable energy over many years, no representatives from the sector could be persuaded to attend the workshops. The ‘absence’ of information in this case is therefore a key result. These big companies simply saw no benefit in participating in research activities. There are many situations in which certain types of stakeholder are routinely marginalized, for example indigenous groups in the case of mineral approvals, drug users in government substance addiction policies, small farmers in the development of agricultural subsidy regimes. Sometimes, as in the case of the example noted above, powerful stakeholders self-exclude, preferring to make their own rules without reference to anyone else. The car industry, it is assumed, is likely to be in favour of a low-carbon future which maintains existing models of car ownership, unless an alternative way of returning equivalent profits to shareholders is assured. Integrative studies in electric vehicle adoption should look for the missing or marginalized stakeholders in this hyperactive discourse, not just listen to governments’ preferred strategies or industry talking points.

A further aspect of the search for representativeness is the issue of methods. In standard disciplinary science, it is usual to write a careful justification of a method used, and then show how the application of the method led to a particular result. There is of course nothing at all wrong with such an approach, it is the basis of modern science. A laboratory which tests for presence of a virus will do such a test routinely and have a well-defined method workflow, which may change hardly at all over years or even decades. Such work may be rigorous and valuable, but it is not integrative. An integrative research approach would look to include several different methods, if they exist and are appropriate, and highlight the possible gaps that might exist, as an intrinsic part of the study, not as a final note under limitations. In social science, an integrative approach implies accounting for information from multiple sources (not just publishing results from a single survey); in statistics, it implies a careful study design which tests different types of approaches, not just using a single favourite statistical test (e.g. [36]). Integrative research approaches, logically, may not be desirable or necessary in every context. It is at the science–policy interface where integrative research is most valuable.

### 3.3. Knowledge exchange mechanisms across the science–policy interface

The emphasis on knowledge exchange between the research community (science) and political actors (policy) has long been of great interest on both sides of this (supposed) divide. Older framings (e.g. [37]) tended to stress economic benefits to business or to the wider economy through the idea of ‘technology transfer’, something that became a central concern of the early literature on socio-technical transitions (e.g. [38]). McKelvey & Henderson [39], despite the title of their piece, were mostly concerned with the question of ecosystem valuation, rather than analysis of the science–policy interface as we would understand it today, but one of the last lines is revealing: 'As scientists, we must be willing to propose ecological priorities. In so doing, we need to recognize explicitly that such prioritization rests ultimately on human value judgments and cannot be justified on purely "scientific" grounds' [39]. In other words, in the context of the impact of air pollution on biodiversity (the topic of their study), the authors acknowledge that science is not a value-free activity, and that scientists should advance their recommendations and preferences even in questions of prioritization, which would seem to belong to the domain of policy. Jones *et al.* [40] take a broader view of the science–policy interface in relation to climate change, examining disappointing experiences in the regulation of acid rain in the 1980s, where scientific results were ignored by decision-makers because they were ‘not timely, clearly connected to policies, and generated with specific policy-related priorities in mind [and because] scientists and policy makers [were] living and working in largely separate environments, with but sporadic connections’ [40]. These authors propose a framework comprising four criteria for translation of climate change research into the policy arena: relevance, compatibility, accessibility, and receptivity.

Bäckstrand [41] applied a post-positivist perspective to understand the science–policy interface in the context of regulations on transboundary air pollution. Post-positivist discourse is very clearly present in the work of Winder [6,7], who defines integrative research as ‘the process of creating knowledge across the boundaries of epistemic communities’ [6]. Though he is mainly concerned with research policy, rather than the implications of that research for environmental management, the logical extension of Winder’s arguments is that humans, both inside and outside of science, belong to different knowledge communities, and that successful integrative research for policy attempts to identify those communities and exchange knowledge between them. This theme is taken up by Stirling [20], writing about participatory appraisal of environmental policy, who introduces the key concept of ‘opening-up’ and ‘closing-down’ mechanisms. For Stirling [20], ‘opening up’ aims to ‘[reveal] to wider policy discourses the detailed implications of different sources of information and the role of different disciplines, divergent social values and conflicting interests in conditioning disparate interpretations of the available evidence’, while ‘closing-down’ activities try to ‘ “assist” decision-making by cutting through the messy, intractable and conflict-prone diversity of views and develop instead a clear authoritative prescriptive recommendation’ [20]. In other words, ‘opening up’ promotes noisy discourse around contentious issues, while ‘closing down’ tries to shut out the noise and make decisions. It is probably not coincidental that it is the most intensely practical of the small nucleus of integrative research papers (table 1), that of Macleod *et al*. [21], that makes one of the strongest contributions to the establishment of integrative research as a robust theory. These authors unify Stirling’s [20] distinction around activities at the science–policy interface as ‘opening up’ or ‘closing down’ with the integrative research framing and show what this means with concrete examples from environmental policy in the UK. They call for opening-up and closing-down mechanisms to be linked as part of an iterative process [21]. Cartledge *et al.* [28] refine these arguments slightly by clarifying that the boundaries between knowledge communities across the policy interface are ‘fuzzy’—research may be done by engineers, scientists may be involved in politics, policymakers are also citizens. True ‘opening-up’ activities involve messy participation with uncertain outcomes. For this reason, Cartledge *et al*. [28] argue ‘All integrative research requires a dynamic trade-off between closing-down and opening-up activities. Any attempt to open problems up creates problems at the science–policy interface, not because regulators are bad people, but because a natural subsidiarity makes opening-up actions seem very disruptive’. Hernandez-Jimenez & Winder [29] elaborate on this with the example of an integrative research project in the Madrid region, Spain, where urban development has been poorly controlled and serves the interests of powerful actors. In this example, the ‘opening-up’ process is well established, but no action has been taken to stop environmental degradation or even illegal development: ‘Key actors pay lip service to governance and facilitate noisy political discourse but do so in such an undisciplined way that no coherent message rises up the administrative hierarchy…badly managed integration can be a very neat way of frustrating innovation’ [29]. The implication of this finding, especially if participation is viewed as instrumental, rather than normative or substantive (*sensu* [20]) is that bad-faith actors can avoid compliance by creating noisy discourse. In fact, as I argue here, both opening-up and closing-down mechanisms at the science–policy interface are susceptible to disruption by powerful actors. Moreover, this is what we are currently seeing, with the failure of climate policy to limit climate warming to within agreed temperature bounds or to propose radical solutions involving genuine societal transformation, e.g. to limit or redistribute resource use [42].

## 4. Related framings: wicked problems and post-normal science

Having explored in some detail the integrative research concept through its original application and literature, I now turn to some closely related concepts in sustainability science. These are ‘wicked problems’ (e.g. [43]) and ‘PNS’ (e.g. [44]). Both of these concepts were present from the beginning in the integrative research literature but, unlike integrative research, seem to have retained their currency in the literature on environmental research for policy. Examination of this literature shows not only that some of the researchers that earlier used integrative research framings later moved to reframe their work to emphasize ‘wicked problems’ or ‘PNS’, but also that the integrative research community in the sense defined by the literature in table 1 seems itself to have disappeared. In this sense, the three concepts of ‘wicked problems’, ‘PNS’ and ‘integrative research’ seem to fit the scenario described in the introduction to this piece, in that they are overlapping, but only incompletely and unsatisfactorily so. At the same time, these three concepts are rarely invoked together, yet it is logical to do so, as the following sections explain.

### 4.1. Wicked problems

The identification of a category of wicked problems can be traced to the foundational paper of Rittel & Webber [9]. These authors, writing from the perspective of social planners of their era, reflected that traditional public planning tasks (e.g. addressing poverty) are intractable since both the issues they aim to resolve and the solutions proposed to resolve them are contested, there is no agreed framework and no optimal outcome. The problems cannot be solved, argue these authors, since they are not problems in the ordinary sense. The literature in this area is quite large, and environmental management problems were identified early on as susceptible to ‘wickedness’ (e.g. [45]). As Duckett *et al*. [43] have noted, even the definition of what constitutes a wicked problem is itself contested. For Miller [46], for example, *wicked* problems are those that cannot be easily bounded and for which conceptual or technical tools are not readily available, as distinct from *trans-science* problems that are amenable to scientific approaches but cannot be resolved due to practical constraints (time and money), or *value-laden* problems where the solution depends on choices and individual valuations made by different stakeholders. Other authors have tended to regard problems of practicability and stakeholder value judgments as intrinsically part of what makes environmental problems wicked. Winder [7] refers to ill-posed problems (a synonym of wicked problems) as those which may not have a unique solution, or that the response differs according to stakeholders’ belief systems. Duckett *et al*. [43] make a significant contribution to the literature on wicked environmental problems by synthesizing the many anarchic and overlapping definitions from earlier literature into six more concise categories—for these authors, wicked problems are: (1) indefinable (and non-generalizable), (2) ambiguously bounded, (3) temporally exacting, (4) repercussive, (5) doubly hermeneutic and (6) morally consequential. They then show how these characteristics have been addressed in practice by land management stakeholders in four discrete wicked problem cases in Scotland. Two of the four cases discussed by Duckett *et al*. [43] merit detailed consideration here.

#### 4.1.1. Mitigating climate change through woodland planting

The question of woodland planting in Scotland as a climate change mitigation activity clearly falls into the category of wicked problem. First outlining why this case qualifies as ‘wicked’, Duckett *et al.* [43] then show how the challenges are approached in practice, e.g. by developing strategies involving multiple agencies (boundary spanning), allowing stakeholders on the ground to make decisions (the subsidiarity principle, implying meaningful participation) and committing to public involvement in decision-making (public participation). Though many hectares of new woodland have been planted since 2015 when Duckett *et al*. [43] were drafting their paper, the issue is now more deeply contested than before. In addition to the food versus forestry conflict identified by these authors, recent studies express concern that overall net carbon gain may be less than supposed, due to carbon emitted from planting, damage to organic soils from new afforestation and the effects of climate change on forest growth [47,48]. Afforestation now has significant momentum and seems unlikely to be halted any time soon. However, if the commitments made by Scottish Government (boundary spanning, subsidiarity, public participation etc.) have been carefully followed through in practice, perhaps more good will have been done than harm.

The case is discussed here because it seems that all of the approaches proposed by Duckett *et al*. [43] as means to address the wicked nature of the problem can be explicitly mapped onto the three key criteria identified in the integrative research literature—the knowledge cycle; representativeness and participation; and knowledge exchange mechanisms. In this case, and admittedly with the benefit of hindsight not available to Duckett *et al*. [43], an integrative research framing might have helped to clarify that the science–policy process was in danger of closing down on an issue that had not really been resolved to the satisfaction of some key stakeholders. Perhaps the knowledge cycle has become a feedback loop between policymakers and businesses, an ideas-free ‘ascientific’ ratchet, where dissenting stakeholders are shut out of the dialogue. Of course, this rather confirms Duckett *et al*.’s [43] inclusion of this question in the category of wicked problems.

#### 4.1.2. Mitigating rural diffuse pollution in freshwater systems

The second of Duckett *et al*.’s [43] cases is of interest here since the example of the Diffuse Pollution User Manual was chosen by Macleod *et al.* [21] as an illustration of a closing-down mechanism at the science–policy interface, developed through a large discipline- and sector-spanning integrative research process. Though Duckett *et al.* [43] and Macleod *et al.* [21] both work for the same institution, the papers were published 8 years apart and have no co-authors in common, so we have the unusual situation of two apparently different approaches to the same problem, in the same location, by two different groups of scientists. The essential point here follows that made above. Duckett *et al*. [43] begin by identifying the wicked characteristics of diffuse pollution control, and showing how they can be addressed with reference to the various solutions identified in the literature, shown on what the authors term the Wicked Wheel (fig. 1 in [43]). But diffuse pollution control practice emerged years earlier through a process of integrative research, and although the chronology is reversed in the two papers, the integrative research approach followed by Macleod *et al*. [21] is clearly a response to the wicked characteristics of the problem that Duckett *et al*. [43] describe. It is easy to see how the approaches described in each of these papers fit together. The opening up of the diffuse pollution problem to multiple cross-sector and cross-disciplinary stakeholders marked the initiation of a knowledge cycle with back-and-forth exchange across the science–policy interface, eventually closing down on the Diffuse Pollution User Manual.

This brief discussion has intended to show that the identification of wicked environmental problems is a key step in an integrative research approach to problem-solving across the science–policy interface. The wicked problem definition exercise demonstrated by Duckett *et al*. [43] shows how to unpack such problems into six wicked components, enabling them to be addressed through structured integrative research activities (e.g. [12,21]).

### 4.2. Post-normal science

PNS is associated with the work of Silvio Funtowitz and Jerome Ravetz, and seems to have emerged in the 1990s, though it is rooted in earlier currents of thought (e.g. [49]). It is mostly defined by what is it not (normal, absolutist, dogmatic, certain, objective etc). Funtowicz & Ravetz [11] offer the following:

This emerging science fosters a new methodology that helps to guide its development. In this, uncertainty is not banished but is managed, and values are not presupposed but are made explicit. The model for scientific argument is not a formalized deduction but an interactive dialogue. The paradigmatic science is no longer one in which location (in place and time) and process are irrelevant to explanations. The historical dimension, including reflection on humanity’s past and future, is becoming an integral part of a scientific characterization of Nature. [11]

Ravetz [50] noted that ‘The insight leading to PNS is that in the sorts of issue-driven science relating to environmental debates, typically facts are uncertain, values in dispute, stakes high, and decisions urgent’. In this sense, PNS seems like an extension of the wicked problems discourse. The frequent references to the environment in the work of Funtowitz and Ravetz has tended to give environmental scientists a sense of ownership of the concept, which is perhaps misplaced, since currents of thought outside of environmental science had already signalled the weakness of ‘normal’ science paradigms and proposed new directions. For example, Ian Hodder’s famous essay entitled ‘Post-processual archaeology’ [51] outlines the inherent subjectivity of interpretations of past human actions and cultures, noting that ‘our cultural paradigms infuse not only our evaluations of the past, but also our evaluations of theories developed in living societies’. For Hodder [51], norms influence process, but are political in nature, thus (social) processes cannot be studied in isolation (hence post-processualism). This dichotomy can only be overcome by placing human agency at the centre of social theory [51]. In a post-normal world, facts are disputed, and values differ, and thus ‘science derived from textbooks must be supplemented by other ways of knowing’ [50]. It is clear from the foundational literature (table 1) that integrative research sits within and is intrinsically part of PNS. According to Winder [7], for example: ‘knowledge is a set of beliefs that exists within a defined community by negotiation and common consent’. Park & Seaton [12] are concerned with decision-making under uncertainty, mirroring the interest in ‘systems uncertainties and decision stakes’ of Funtowicz & Ravetz [11]. Tress *et al*. [8] talk of bridging ‘knowledge cultures’ and propose to ‘judge results in relation to existing beliefs and commonly held attitudes’. Both Hernandez-Jimenez & Winder [29] and Hewitt *et al*. [32] are fundamentally concerned with power dynamics in the stakeholder community and how they frustrate constructive action to implement environmental policies and goals.

Waylen *et al*. [44] also note similarities between the context of PNS and the wicked problems framing but lament the relative scarcity of experiences in the application of PNS to environmental challenges. Though this does seem broadly to be the case, it is also likely, as I have tried to show in the previous paragraphs, that PNS concepts and worldviews are hidden inside studies that do not explicitly make the connection. It may be that the lack of explicit mention of the concept does not mean that it is not deeply embedded in modern thinking on socio-environmental systems. At the same time, these authors’ observations do clearly point to a worrying tendency, and their study, which reflects on experiences from scientists working with a wide range of policy stakeholders on critical environmental issues, is timely. Why, we can ask, is integrative research (for this is surely what Waylen *et al*. [44] are engaged in) still so difficult?

Recent work on wicked problems in environmental science explicitly connects to activities that are part of the integrative research approach. The wicked problems perspective as applied to policy by Duckett *et al*. [43] could profitably be linked to the science–policy mechanisms framing of Stirling [20] and Macleod *et al*. [21], allowing us to detect when the cycle had switched to closing-down mode too early, as may have been the case with afforestation for climate mitigation in Scotland (and probably elsewhere). Though reopening the problem for consultation might be tiresome to some, it is important not to end up arguing that it is better to do the wrong thing quickly than waste time trying to find out how to do the right thing. If planting trees actually does have a net carbon benefit everywhere it is proposed to put them, then the case is closed, and more trees should be planted. The problem is that it is now no longer certain that it does [47,48]. If environmental research across the science–policy interface were understood as a cycle, not a one-way street, these kinds of problems might be avoided. The integrative research framing makes this cycle very clear, other framings, unfortunately do not.

On the other hand, PNS at the science–policy interface, as described by Waylen *et al*. [44], is virtually synonymous with integrative research. However, as with wicked problem-framings, there is a conceptual weakness in that the key elements of the integrative research process are not always explicit, and as such it is hard to link participatory knowledge exchange processes with specific outcomes. In many deliberative exchanges, outcomes may not be those expected; nevertheless, the integrative research framing helps clarify how and why this might have occurred.

## 5. Concluding discussion: why we need integrative research

What I have tried to show in this piece is that, while we cannot of course be completely sure ‘who killed integrative research’, the closely related wicked problems and PNS framings have mostly overtaken the integrative research concept in current usage in the environmental policy literature. The wicked problems framing is mostly a critical discourse about problem definition (very effectively operationalized by Duckett *et al*. [43]) while PNS is a necessary and valuable critical reaction to past excesses and scientific hubris. Yet in an increasingly contested and fractious environmental policy context, where vocal arguments are advanced by powerful groups in opposition to radical action, both these discourses may seem more acceptable than integrative research. A wicked problems framing may be (mis)understood as implying that environmental problems are too difficult to solve, while PNS perspectives may appear unthreateningly abstract and action-light.

**Figure 2.**
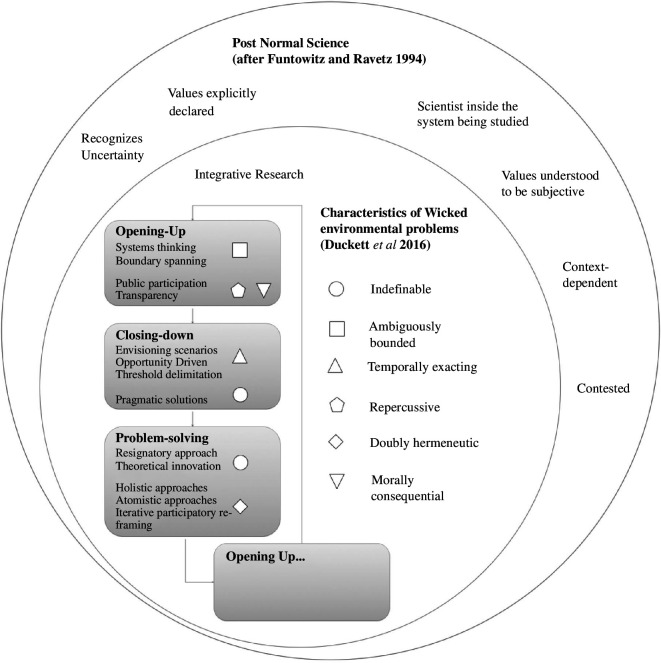
Integrative research for wicked environmental problems (after Duckett *et al*. [43]) in the PNS universe (after Funtowitz & Ravetz [11]).

Integrative research, however, actually provides a specific template for action. It shows how to structure research activities that cut across different knowledge communities. A ‘Russian doll’ structure can be envisaged, in which integrative research is the child of PNS, allowing us to tackle wicked problems (figure 2). Each of these three concepts needs the other, and that by integrating them, we can do good work that might otherwise be left undone.

Of the three key concepts that I have argued are central to integrative research at the science–policy interface (the knowledge cycle, representation and participation and knowledge exchange mechanisms), only representation and participation are convincingly replicated in wicked problems or PNS framings. This is encouraging, since without meaningful participation, progress on any issue will grind to a halt, and just deliver the outcome best-suited to the most powerful stakeholder.

But the loss of the knowledge cycle concept is a serious deficiency. Not only does it help us see when processes are stuck and need to move on to the next stage (figure 1), it also encourages us to ask who is involved at different points in the cycle and what form of knowledge construction is being pursued. Stirling [20] has framed this as a dichotomy between discursive (participatory) and analytical (non-participatory) actions. Hernandez-Jimenez & Winder [29] took this one step further and allied discursive research modes with problem-framing and analytical modes with problem-solving. But might not some participatory processes actually deliver analysis, not just discourse? And might not some non-participatory activities contribute to discourse? The framing of course, is too simple. Yet the point of it, something that is entirely lost when we abandon the knowledge cycle concept, is to scrutinize carefully what kind of information is being fed into the system and how it is being created. In this sense, we can see that not every kind of knowledge cycle even need involve science, in the widely understood ‘public good’ sense at all [52]. If global focus alights on just a few techno-solutions (more renewables, electric cars), and large multinationals control ever larger reserves of capital, research divisions of companies like Amazon and Space-X become increasingly powerful, attracting the brightest minds and the largest research budgets. Public universities and research institutes can be eliminated from the research cycle, increasingly relegated to education of the specialized workforce these large companies require. Many elements of the scientific process—publication, to take one example—were long ago surrendered to the private sector, could research not follow too? What is to stop the ivory tower becoming a tower of glass and steel, where the orientation of research and the kinds of problems it is to solve are decided by corporate interests in the boardroom? We could argue that, at least in the English-speaking rich world, such a transformation has already occurred. In the words of Hall [53] ‘The University is being explicitly restructured for the production, circulation and accumulation of value, materialised in the form of rents and surpluses on operating activities’.

In this way, we can see the knowledge cycle concept as an essential safeguard, allowing us to effectively analyse what kinds of information, and which actors are feeding the system. If it is just a closed loop (an ‘ascientific’ ratchet) between a large company and its employees, or wealthy donors and tame universities, dissident perspectives and challenging innovations will be squeezed out, leaving only those ideas which are least threatening to the most powerful groups in society.

With regard to the other missing piece ‘knowledge exchange mechanisms’, Macleod *et al*. [21] have convincingly argued, following Stirling [20], that opening-up and closing-down mechanisms are fundamentally part of the operational structure of integrative research at the science–policy interface. The loss of integrative research framings implies the loss of these mechanisms, or at least, the lop-sided application of just one of the two mechanisms. The connection with climate policy was already made by these authors, who note the work of the Intergovernmental Panel on Climate Change as ‘an established international example of a closing-down mechanism’ [21]. The failure of climate policy to provide inclusive, imaginative solutions that include all stakeholders can be seen as due to the design of the COP process as a ‘closing-down’ mechanism. The disappearance of the integrative research discourse from the environmental policy literature has meant that this key observation has not had the exposure it deserves. Viewed through this lens, we can understand the remarkable persistence of headline climate ‘solutions’ like afforestation (rather than regeneration and ecosystem restoration), electric vehicles (rather than fewer vehicles) and low-carbon buildings (rather than effective development control). It is not just that all of these favoured solutions are business friendly and involve adding new things (though they are), but that the entire system of climate change mitigation is one giant mechanism for ‘closing down’—in other words, excluding dissident stakeholders and bashing out ‘solutions’ that accept the world as it is, rather than changing the course of history through ‘opening up’.

Opening-up processes in environmental policy do of course have their limitations, as the example of Madrid’s real estate bubble shows [29]—it is quite easy for powerful actors to generate the outcome they prefer (usually the status quo) just by creating noise, for example by endlessly filing claims and counter claims, proposing new laws and modifications, insisting that every stakeholder should be present at every meeting and failing to clearly identify who is responsible for implementation [54]. But in these cases, we return to the recommendations of Macleod *et al*. [21]—that opening-up and closing-down mechanisms must be linked, and they must be iterative. Thus, bad-faith negotiations that are endlessly prolonged must be driven towards a solution (closing down), but when solutions proposed are all of the same type, we must accept the need to stop trying to close down and engage a much wider participant group (opening up). It is integrative research, operationalized through the three guiding principles exposed in this piece, that shows the way forward. The poverty of discourse on environmental solutions beyond business as usual (building, planting, buying something) and blatant hijacking of the international climate policy process by fossil fuel interests, signals the desperate need for new ways of thinking about these pressing problems.

To fully adopt these recommendations for environmental research at the science–policy interface requires some operational adjustments to the currently accepted practice of science. These can be seen to point in quite radical directions. One approach would be to remove the separation between research and policy by making environmental protection agencies fully responsible for carrying out the research that addresses their problems, rather than acting as hands-off enforcers or commissioning bodies. More radically, research institutions could be given agency competencies, such that, when their own research indicates that action should be taken, they would be expected to move seamlessly to take it, rather than step back and offer advice. By blurring the boundaries between research and action, sectoral separations are transgressed such that researchers are no longer expected to inform without acting and neither policymakers nor regulators to act without information. Equally, institutional restrictions on research funding could be relaxed, such that citizen groups with proven competences are eligible for certain research streams. European research funding, for example, is mainly destined to universities and large research institutes, followed by larger private companies. Small not-for-profit organizations, citizen groups and individuals are excluded in practice, if not by design.

The type of research which is publicly funded would also need to be radically transformed. For example, Overland & Sovacool [55] in their analysis of global funding for climate change, found that natural and technical sciences received disproportionately more funding than the social sciences over the same time period. As these authors state: ‘The funding of climate research appears to be based on the assumption that if natural scientists work out the causes, impacts, and technological remedies of climate change, then politicians, officials, and citizens will spontaneously change their behaviour to tackle the problem’ [55]. Small wonder, then, that reflective, socially transformative approaches to the most pressing problems of our time, the climate and environmental crises, seem lost in a sea of business-as-usual techno-solutions, which, to judge by their results, are mostly not solutions at all.

Finally, in the words of Park & Seaton [12] ‘No attempt is made to suggest that there is a single method of carrying out research toward a sustainable systems framework’. At the same time, without any framework at all, it is easy to lose sight of key goals or misunderstand important interactions. The contention in this paper is that the loss of the integrative research framing is a real loss, not just a simple change of emphasis. We should seek to recover it.

## Data Availability

The paper draws only on data from published literary sources.

## Footnotes

^1^
‘Disciplines are fixed, governable, institutionalized conventions, while knowledge communities are flexible, dynamic, often subversive and sometimes viciously competitive’ [7, p. 76].
